# Co-Transplantation of Endothelial Progenitor Cells and Pancreatic Islets to Induce Long-Lasting Normoglycemia in Streptozotocin-Treated Diabetic Rats

**DOI:** 10.1371/journal.pone.0094783

**Published:** 2014-04-14

**Authors:** Paola Quaranta, Sara Antonini, Saturnino Spiga, Benedetta Mazzanti, Michele Curcio, Giovanna Mulas, Marco Diana, Pasquina Marzola, Franco Mosca, Biancamaria Longoni

**Affiliations:** 1 Department of Translational Research and New Technology in Medicine, University of Pisa, Pisa, Italy; 2 Department of Life and Environmental Sciences, University of Cagliari, Cagliari, Italy; 3 Department of Clinical and Experimental Medicine, Haematology Section, University of Florence, Florence, Italy; 4 U.O. Immunohaematology, Azienda Ospedaliera-Universitaria Pisana, Pisa, Italy; 5 Department of Chemistry and Pharmacy, “G. Minardi” Laboratory of Cognitive Neuroscience, University of Sassari, Sassari, Italy; 6 Department of Computer Science, University of Verona, Verona, Italy; Université Paris Descartes, France

## Abstract

Graft vascularization is a crucial step to obtain stable normoglycemia in pancreatic islet transplantation. Endothelial progenitor cells (EPCs) contribute to neoangiogenesis and to the revascularization process during ischaemic events and play a key role in the response to pancreatic islet injury. In this work we co-transplanted EPCs and islets in the portal vein of chemically-induced diabetic rats to restore islet vascularization and to improve graft survival. Syngenic islets were transplanted, either alone or with EPCs derived from green fluorescent protein (GFP) transgenic rats, into the portal vein of streptozotocin-induced diabetic rats. Blood glucose levels were monitored and intraperitoneal glucose tolerance tests were performed. Real time-PCR was carried out to evaluate the gene expression of angiogenic factors. Diabetic-induced rats showed long-lasting (6 months) normoglycemia upon co-transplantation of syngenic islets and EPCs. After 3–5 days from transplantation, hyperglycaemic levels dropped to normal values and lasted unmodified as long as they were checked. Further, glucose tolerance tests revealed the animals' ability to produce insulin *on-demand* as indexed by a prompt response in blood glucose clearance. Graft neovascularization was evaluated by immunohistochemistry: for the first time the measure of endothelial thickness revealed a donor-EPC-related neovascularization supporting viable islets up to six months after transplant. Our results highlight the importance of a newly formed viable vascular network together with pancreatic islets to provide *de novo* adequate supply in order to obtain enduring normoglycemia and prevent diabetes-related long-term health hazards.

## Introduction

Pancreatic islet transplantation is a widely accepted therapy for the cure of insulin-dependent diabetes mellitus (IDDM) [1], [2]. Compared to solid organ transplantation, it offers some advantages: low invasive surgery and low incidence of peri-operative risks. Pancreatic islets have a peculiar micro vascular system, known as the insulo-acinar portal system [3]–[5], which is largely destroyed during the isolation procedure, thus requiring rapid revascularization to preserve its performance in the transplant. In the whole pancreas transplantation the anastomosis of blood vessels can lead to rapid revascularization [6], with vessel density and oxygen tension being one and half times that of pancreatic islets [7]–[12], thereby suggesting that reduced oxygen supply may lead to impaired islet function [11]–[13]. After transplantation islets receive nutrients and oxygen only by diffusion mechanisms whatever the implantation site and after one month islets are still not yet fully revascularized [7]. Carlsson *et al*. compared blood perfusion and oxygenation of transplanted islets in three different sites (kidney capsule, liver and spleen) showing that though the three implantation organs differed markedly in their blood perfusion, the islet graft blood perfusion and oxygen pressure was similar irrespective of the implantation site. This suggests that the intrinsic properties of the transplanted islets are more important than the choice of the implantation site [7].

These findings highlight the need for an efficient vascular bed to provide adequate support to the grafted islets. Patients with diabetes are widely affected by endothelial dysfunction as well as cardiovascular disease and an impaired angiogenic response to ischaemic events [14]. As a consequence, the recipients' graft loss cannot be avoided without a suitable therapy to enhance islet revascularization.

Several studies performed on animal models of ischaemic diseases, (including myocardial infarction, stroke and peripheral arterial diseases) showed that neovascularization can be attributed to bone marrow (BM)-derived EPCs highlighting how EPCs can be considered key players in the vascular system for therapeutic potential [15]–[20]. The endothelium repair is assigned to resident endothelial cells in addition to EPCs, which are circulating precursors of adult neovasculogenesis and vascular homeostasis, also involved in the revascularization of injured tissue and tissue repair [21]. They are mobilised to the peripheral blood upon stimuli, including tissue ischaemia and the local release of cytokines and growth factors [22]. EPCs stimulate endogenous angiogenesis by secreting a variety of angiogenic growth factors during the wound healing process [23], [24]. Thus, the whole of the literature suggests that neovascularization around grafted islets has a main role in graft survival and long-lasting functional recovery.

Hess *et al.* reported that transplantation of bone marrow-derived ECs improved hyperglycemia in diabetic mice by contributing to the recovery of injured β-cells and the endogenous beta cell function [25]. Later Mathews *et al.* showed that EPCs are recruited to the pancreas in response to islet injury by inducing a pancreas neovascularization [26].

Accordingly, Kang *et al.* reported that rapid revascularization by co-transplanted EPCs in nude mice yielded a better islet engraftment and a consequent functional recovery with normoglycemia lasting up to 35 days [27].

Overall these results suggested that co-transplantation of pancreatic islets and bone marrow-derived EPCs could be a potential way to improve islet vascularization and overcome the graft loss.

Here we show the development of a novel therapeutic approach to restore islet vascularization after portal vein transplantation and improvement of enduring graft survival by exploiting the angiogeneic properties of the EPCs.

## Methods

### Experimental animals

Inbred male Lewis rats, weighing 275–300 g, (Charles River, Calco, Italy) were used as donors and recipient of the grafts. The animals were fed on standard rodent chow (Rieper, Bolzano, Italy) and water ad lib, and were kept under a 12 h light/dark cycle. Rats were made diabetic by streptozotocin (STZ, Sigma-Aldrich, Milano, Italy, 65 mg/Kg i.p. injection). Blood glucose concentration was determined by means of a commercially available human glucose meter (Stat Strip Xpress, Nova Biomedical UK). Animals with fasting blood glucose higher than 16.7 mmol/l (>300 mg/dL) on at least two consecutive measures were considered diabetic while rats that remained lower than 16.7 mmol/l after 1 week were withdrawn from the study. All the experimental procedures were carried out with the approval of the ethical committee for animal experimentation of the University of Pisa.

### Islet isolation and culture

Pancreatic islets were isolated from rats by collagenase P (Roche Diagnostics, Milano, Italy) perfusion [28] and purified by continuous-density Ficoll gradient. Briefly, the pancreas was distended by bile duct injection of 15 mL 4°C-cold 1 mg/mL collagenase P (Roche Diagnostics) diluted in HEPES-buffered Hank's balanced salt solution (HBSS) (Sigma-Aldrich), and then it was excised and minced. Islets were digested at 37°C for 20 min under constant agitation. Islets were separated from exocrin tissue by centrifugation on a Histopaque (Sigma-Aldrich) discontinuous gradient, were removed from the interface of the layers, were washed in HBSS, and finally resuspended into 10 mL of RPMI (Eurobio, Milano, Italy) supplemented with 10% fetal calf serum (FCS) (Eurobio), 1% L-glutamine (Eurobio), 10 mM glucose (Sigma-Aldrich), penicillin (50 U/ml, Eurobio), streptomycin (50 µg/ml, Eurobio), amphoterycin B (0,2 µg/ml, Eurobio) and 1% HEPES buffer (Sigma-Aldrich) in free floating culture flask. Islets were handpicked under an inverted microscope under sterile conditions and purity was assessed by Dithizone staining (Sigma-Aldrich). For each graft, the total islet mass, expressed as the 150 µm diameter islet equivalent (IE) which was calculated based on volumetric assumptions.

Pancreatic islets were incubated at 37°C (95% air and 5% CO_2_) for 1–2 days before transplantation.

### EPC isolation and expansion

Bone marrow was harvested from both femurs and tibias of Lewis and Lewis *LEW-Tg (EGFP) F455/Rrrc* (RRRC, University of Missoury) male rats, according to Dobson's procedure [29]. Briefly, mononuclear cells (MNCs) were obtained by density gradient centrifugation. Cells were then seeded in 6 well plates coated with 1% gelatin (25×10^6^ MNC/well), cultured in EGM-2-MV BulletKit (Lonza, Milano, Italy) complete medium, (i.e. EBM-2 medium supplemented with 10% FBS, Hydrocortisone 0,2 ml, hFGF-B 2 ml, VEGF 0,5 ml, R^3^-IGF-1 0,5 ml, Ascorbic Acid 0,5 ml, hEGF 0,5 ml, GA-1000 0,5 ml, Heparin 0,5 ml). Cells were incubated at 37°C in a fully humidified atmosphere containing 5% carbon dioxide. On day 4 the medium was replaced and afterwards it was changed every 3 days. EPC colonies appeared in cell cultures after 1 week and cells were then identified by phase contrast microscopy (Laborlux S microscope Leitz, Wetzlar, Germany) as circumvented monolayers of cobblestone-like cells.

### Flow cytometry

Flow cytometric analysis was performed on cells at passage P2 using the following surface antibodies: CD45, CD11b (in order to quantify hemopoietic–monocytic contamination), CD44, CD90, CD31, Endothelium (AbD Serotec, UK; BD Pharmingen, San Diego, CA, USA), CD133 (Miltenyi Biotec, Germany), CD34 (BD Biosciences) and KDR (R&D system, MY, USA). Non-specific fluorescence and morphologic parameters of the cells were determined by isotype-matched mouse monoclonal antibodies and 7-Amino-Actinomycin D (7-AAD) was used to exclude dead cells. For GFP+ EPCs, green fluorescence intensity was assessed at different passages in culture. The cells were acquired with a FACSCalibur (Becton Dickinson, Milano, Italy; argon laser source, with wave length of emission at 488 nm and power of emission 15 mW) and data was analysed by CELL QUEST software (Becton Dickinson).

### Dil-Ac-LDL uptake

To identify *ex vivo* expanded EPCs, the cells were imaged for their incorporation of acetylated low-density lipoprotein (aLDL) labelled with fluorescent Dil dye (Dil-Ac-LDL, Invitrogen, Milano, Italy), Endothelium FITC (AbD Serotec, UK; BD Pharmingen, San Diego, CA, USA) and DAPI (Invitrogen). The cells were incubated with 10 µg/ml Dil-Ac-LDL for 4 hours at 37°C on a glass slide; subsequently, cells were washed and Endothelium was added (10 µg/ml) for 20 min in the dark. After fixation with 10% formalin for 20 min at 4°C, EPCs were washed and DAPI was added following manufacturer's instruction. Cells were visualized by fluorescence microscopy on a Laborlux S microscope (Leitz, Wetzlar, Germany).

### *In vitro* Angiogenesis assay

BD Matrigel Basement Membrane Matrix (BD, Bioscience, Milano, Italy) was thawed on ice overnight, and a volume of 50 µL, added to EGM-2 (Lonza) supplemented with 1% FCS, was spread evenly over each well of a 24-well plate. The plates were incubated for 30 min at 37°C to allow the Matrigel to form a gel. GFP+ EPCs were seeded (3.0×10^4^ cells/cm^2^) in darkness and cultured in 1 ml of EGM-2 medium supplemented with 1% FCS. After 2 hours of incubation at 37°C, phase contrast (bright field) and fluorescence images were recorded on a Carl Zeiss Microscope (Carl Zeiss, Milano, Italy) equipped with a fluorescence camera, (AxioVision 4.8.2 software). Cells were checked approximately every 2 hours to observe the tube formation up to a final time point of 20 hours (Figure S1).

### *In vivo* experiments

The effect of EPC intravenous administration was assessed in a syngenic model (from Lewis donors to Lewis recipients) of a marginal mass pancreatic islet transplantation. Diabetic-induced rats were transplanted into the portal vein with 700 syn-IE alone (n = 6), 700 syn-IE plus 5×10^5^ EPCs (n = 11), or 5×10^5^ EPC (n = 4) into the portal vein.

### Portal Islet Transplantation

Five days after STZ treatment, diabetic rats were anaesthetized (Zoletil 100: tiletamin 90 mg/Kg and zolazepam 10 mg/Kg, i.p, Virbac, Milano, Italy) and transplanted as previously described [30]. Freshly detached 5×10^5^ EPCs were suspended in 500 µl PBS saline buffer solution and concomitantly mixed with 700 syn-IE freshly isolated. The cell mix was then incubated at 37°C 5% CO_2_ for up to one hour by giving gentle pats to the mix holder every 15 minutes to avoid adherence to the culture plastic. 700 syn-IE alone or 5×10^5^ EPCs plus 700 syn-IE were finally injected through a 26G needle and re-flushed 2–3 times into the portal vein of each animal. To prevent potential experimental biases, the aliquots from the same batch of islets were transplanted in both experimental groups in parallel each time.

### Assessment of Graft Function

Fasting blood glucose levels were used to assess islet graft function. The measures were performed at defined time points from transplantation. Graft failure was defined as a return to hyperglycemia >16.7 mmol/l (300 mg/dL) by two consecutive measurements.

### Intraperitoneal glucose tolerance test (IPGTT)

Rats were given an intraperitoneal glucose tolerance test at different time point from transplantation (15, 30 and 180 days). After fasting overnight the animals were injected with 2 gr/Kg body weight of glucose intraperitoneally. Blood glucose was measured at 0, 15, 30, 60, 90, 120 and 150 minutes after injection. These values were then compared to the control values (diabetic and healthy rats).

### Real-time PCR

Liver samples were collected to assess the mRNA expression of all genes involved in vascularization. Since transplanted islets are homogeneously distributed throughout the whole liver [31], a random sampling of the tissue in six different points for each animal was performed (not exceeding 30 mg per sample). The experiments were run in triplicate. Total RNA extraction and purification of EPCs/GFP+ EPCs and RNAlater-treated liver samples of transplanted animals were carried out using the RNeasy Mini kit (Qiagen, Hilden, Germany), according to the manufacturer's protocol. The On-Column DNase I enzymatic digestion set was used to remove genomic DNA from total RNA preparations. The amount of extracted RNA was quantified by measuring the absorbance at λ = 260 nm with a Biophotometer Plus Eppendorf (Eppendorf, Milano, Italy) spectrophotometer. Reverse transcription of the samples was performed with RevertAid First Strand cDNA Synthesis Kit (Fermentas, Thermo Scientific, Milano, Italy). The VEGF-A, PECAM-1, HGF and ANG-1gene expressions were evaluated using a SYBR Green Master Mix technique (Qiagen), according to the manufacturer's instructions. Controls and samples were run in triplicate on 96-well reaction plates with the iQ5 Multicolor Real-Time PCR Detection System (Bio-Rad, Milano, Italy).

For all pairs of primers the following experimental run protocol was used: denaturation program (95°C for 3 min), amplification (95°C for 15 s, 62°C for 1 min, 40 repetitions) and quantification program (melting curve program at 56–95°C with a heating rate of 0.5°C per second and continuous fluorescent measurement). Primers (see table 1) were generated according to published protocols [32]–[36].

**Table 1:** Sequence of the primers used in Real-time PCR.

Gene	Primer sequences
PECAM-1 (F)	TCAGCTGCCAGTCAGTAA ATGG
PECAM-1 (R)	TCTGGAAGTTGCTCTTTGCTCTT
VEGF-A (F)	GAGGAAAGGGAAAGGGTCAAAA
VEGF-A (R)	AATCCTGGAGCGTTCACTGTG
ANG-1 (F)	GTGGCTGGAAAAACTTGAGA
ANG-1 (R)	ACATCCCGTCTTGAAATCCA
HGF (F)	CTTCTGCCGGTCCTGTTG
HGF (R)	TCTTCTCTTCTTCTGTCCTTCTGC
GAPDH (F)	GTATTGGGCGCCTGGTCACC
GAPDH (R)	CGCTCCTGGAAGATGGTGATGG

All the amplifications were carried out with normalization of gene expression against the glyceraldehyde 3-phosphate dehydrogenase (GAPDH) reference gene. Estimation of gene expression was calculated according to the 2^-ΔΔCT^ method using the Bio-Rad iQ5 2.1.

### Histology

Rats were transcardially perfused by 200 ml of PBS at 0°C followed by 400 ml of 4% paraformaldehyde with a pH of 7.4. Liver samples were post-fixed in the same solution for 12 hours at 4°C and, after washing, were transferred in a 30% sucrose solution in PBS for cryoprotection for 2–3 days. Afterwards the liver samples were cut in 16 µm thick slices using a cryostat (Microm Cryo-Star HM 560). In order to detect sparse pancreatic islet in liver samples, sections were collected in PBS and one in every six was stained by hematoxylin and eosin (H&E). Slices were rinsed in PBS (3×10 min) and pre-incubated in 5% normal goat serum (NGS) (Invitrogen) containing 5% bovine serum albumin (BSA) (Sigma-Aldrich) and 0.3% Triton-X-100 (Sigma-Aldrich) in PBS overnight at 4°C and then incubated, in various combinations, with the following primary antibodies:

1. mouse anti-PECAM-1 (TLD-3A12) (Santa Cruz Biotechnology, Heidelberg, Germany), primary antibody (1∶200), overnight in PBS 4°C.
2. rabbit anti-insulin (Abcam, Cambridge, UK) (1∶200) for 48 hours in PBS 4°C.

Secondary antibodies:

1. Donkey Alexa Fluor 594 (Invitrogen) anti mouse (1∶200) for 4 hours in PBS at room temperature.
2. biotinylated anti rabbit (Vector Laboratories, Peterborough, UK) (1∶200) for 4 hours in PBS at RT and Fluorescein-Streptavidin (Vector Laboratories) (1∶200) in PBS for 4 hours at room temperature.

All slices were washed 3×10 min in PBS and covered with a Vectashield cover-slip (Vector Laboratories). Control sections were treated with secondary antibodies as described above, without primary ones. Slice analysis was performed using a Leica 4D confocal laser scanning microscope with an argon–krypton laser (Leica, Milano, Italy). Confocal images were generated using PL Fluotar 10X (na.0.3), 40X oil (na.1.00) and 100X oil (na.1.3). Surface rendering was used to display and analyze the structures creating shaded solid bodies. The rendered surfaces were interactively displayed and analyzed for global structure properties and interaction between stained elements. ImageJ 1.43, and Bitplane Imaris 7.4.2 were used to visualize and count maximum intensity, average intensity and co-localization.

### Statistical analysis

Data was analyzed using GraphPad Prism version 5 software and all numerical values were expressed as mean ± SEM. Data was compared using one way ANOVA followed by Tukey's test to assess statistical significance. A value of *p*<0.05 was considered significant.

## Results

### Characterization of EPCs

**Figure 1:**
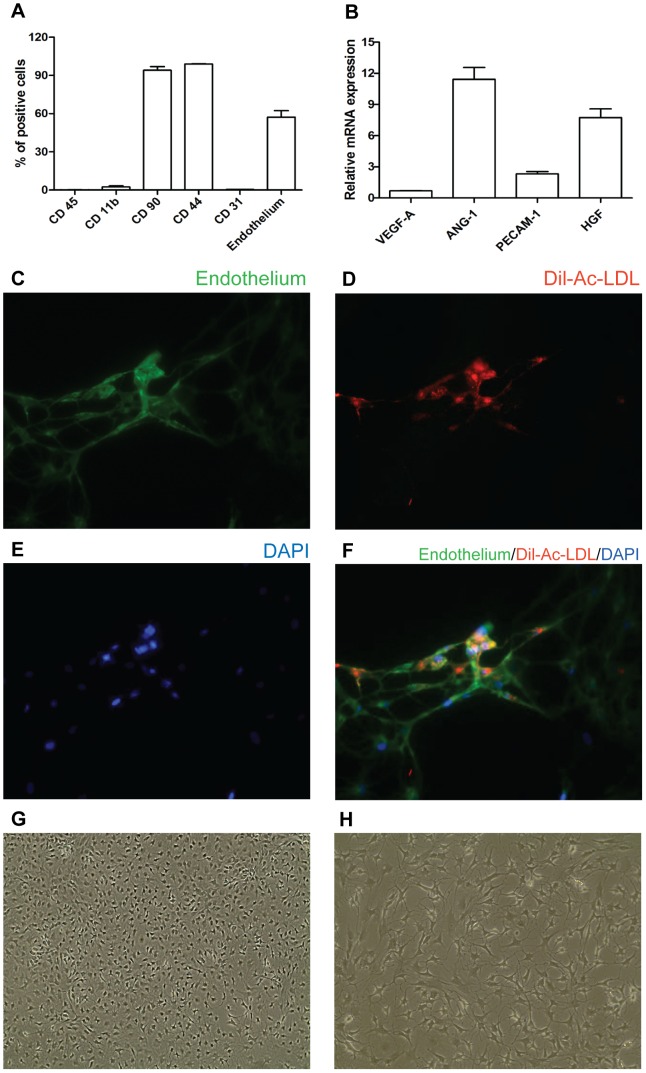
Characterization of EPCs. Flow cytometric analysis of EPCs. (A) Percentage of expression of CD45, CD11b, CD90, CD44, CD31 and Endothelium markers were shown. Each column represents mean ± SD of six different clones. (B) mRNA levels of VEGF-A, ANG-1, PECAM-1 and HGF in cultured EPCs (***p<0.0001 VEGF-A versus ANG-1; ***p<0.0001 VEGF-A versus HGF; ***p<0.0001 ANG-1 versus PECAM-1; *p<0.05 ANG-1 versus HGF; ***p<0.0001 PECAM-1 versus HGF). Error bars are ± SEM. (C-F) In vitro functionality of EPCs was identified by positive staining for endothelial antibodies (Endothelium (C, green), Dil-Ac-LDL (D, red), nuclei (E, blue), merge (F)).(G, H) EPCs cobblestone-like morphology.

EPCs isolated and expanded from BM of male Lewis and Lewis EGFP rats were analysed by morphology, flow cytometry and Dil-Ac-LDL uptake (Figure 1). Since wild type and GFP+ EPCs showed comparable characteristics, data were reported as mean ± SD of the different analysed clones. EPCs were positive for CD44, CD90 and Endothelium (ox43 protein expressed on vascular endothelial cells in each rat tissue), while negative for CD31, typical of mature endothelial cells, and for hematopoietic markers, (CD45, CD11b) (Figure 1A). We did not detect significant amount of positive cells for CD133 (1.5±1.7, mean±SD), CD34 (0.6 ± 0.8, mean±SD) and KDR (0.3±0.4 mean±SD) even though some authors have previously reported converse results [37]. GFP+ EPCs showed a GFP expression over 94% at every passage along with the presence of endothelial markers (data not shown). RNA was extracted by *in vitro* generated EPCs to study the gene expression of PECAM-1 (CD31), VEGF-A, ANG-1 and HGF by real-time PCR. Data showed high expression of ANG-1 and HGF, in contrast, a minor expression of PECAM-1 and a negligible level of VEGF-A were observed (Figure 1B). The functional characterization of EPCs showed the property of the cells to uptake Dil-Ac-LDL (Figures 1C–F). I*n vitro* angiogenic ability of EPCs was assessed (both in fluorescence and in bright-field) within the first 12 hours after seeding, by using an endothelial tube-formation assay. At various time points EPCs are shown to grow into an organized capillary network (Figure S1) and revealed a classical cobblestone-like cell morphology (14 days from seeding) (Figures 1G–H) [38].

### Long-lasting glycaemic control in animals receiving EPCs and islets

**Figure 2:**
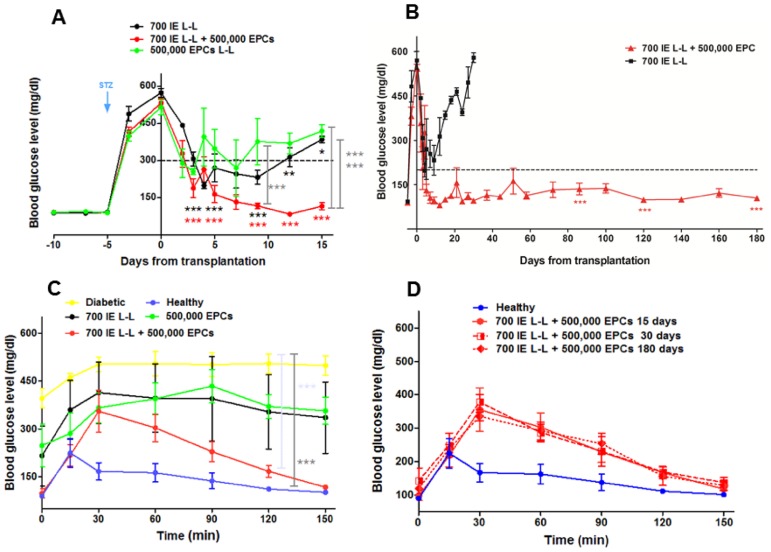
Effect on the glycemic levels of cell-based treatments. Glycemic levels of 700 syn-IE + 500,000 EPCs (n = 11, red line) compared with 700 syn-IE (n = 5, black line) and with 500,000 EPCs alone (n = 4, green line). (A) Co-transplantation of 700 syn-IE+500,000 EPCs induced a faster decrease of blood glucose levels than the other treatments (***p<0.0001, **p<0.001; *p<0.05). One way ANOVA was carried out to compare different treatments (***p<0.001 700 syn-IE+500,000 EPCs versus 700 syn-IE; ***p<0.01 700 syn-IE+500,000 EPCs versus 500,000 EPCs alone). On day 15^th^, the difference between 700 syn-IE+500,000 EPCs and 700 syn-IE was extremely significant (*** p<0.001). (B) Monitoring of blood glucose levels in the long-term showed that animals receiving 700 syn-IE+500,000 EPCs maintained normoglycemic values for the overall observation time, (6 months) compared to animals treated with 700 syn-IE alone (***p<0.001). (C) IPGTT in transplanted animals. Glucose clearance in the transplanted groups on the 15^th^ post transplantation day. The comparison of glucose clearance for animals receiving 700 syn-IE (black line), 500,000 EPCs alone (green line) and 700 syn-IE+500,000 EPCs (red line) with healthy (blue line) and diabetic animals (yellow line) showed a return to normoglycemia within 120 min since the glucose injection only for the co-transplanted and healthy groups. One way ANOVA to compare different treatments, showed that at 60, 90, 120 and 150 min. healthy and co-transplanted groups showed no statistically significant differences, while the two groups showed statistically significant differences compared to the diabetic group (***p<0.001). (D) Glucose clearance in the co-transplanted group at different time points. The glucose clearance was measured at 15, 30, 90 days after transplantation for animals receiving 700 IE+500,000 EPCs. One way ANOVA showed that the differences among the curves were not statistically significant. Error bars are +/− SEM. One way ANOVA was performed within each curve by comparing different time points versus day 0.

Based on our previous results [30] we decided to co-transplant EPCs (5×10^5^) with a marginal mass of 700 syngenic islet equivalent (syn-IE) into the portal vein of streptozotocin-induced diabetic rats. The same dose of EPCs was found to be effective also by other authors [26], [27], [38]. EPCs derived from GFP-transgenic rats were used to follow the fate of these cells after their injection and to evaluate their contribution to the formation of new vessels in the graft. Both EPCs and GFP+ EPCs were used in association with pancreatic islets to perform transplants; since glycaemic curves were comparable without significant differences in blood glucose levels, all data were grouped and plotted together. Blood glucose levels of transplanted animals were monitored for up to 6 months. The syngenic transplantation of 700 syn-IE alone (n = 5) induced a fast decrease in blood glucose levels, which raised to high values within twelve days of observation. A gradual recurrence of diabetic levels was observed up to day 15 (Figure 2A). Co-transplantation of 700 syn-IE and 500,000 EPCs (n = 11) showed a faster decrease in blood glucose levels compared to animals receiving 700 syn-IE alone and the values remained under the diabetic threshold throughout the whole observation time (p<0,0001 Figures 2A and B). In contrast, administration of a single dose of 500,000 EPCs alone (n = 4) did not affect glycemia of transplanted animals (Figure 2A). Although a minor decrease in blood glucose levels was observed at day 3, the change was not significant. The animals receiving EPCs or 700 syn-IE alone were monitored for 30 days after transplantation to confirm their diabetic status; during this time lapse (between 15 and 30 days after transplant) the animals appeared very weak, their body weight decreased and we did not observe a recovery from their diabetic status, so the animals were sacrificed (Figure 2B). Conversely, the group of animals co-transplanted with 700 syn-IE plus 500,000 EPCs maintained normoglycemia for 180 days. The response was striking: blood glucose levels were under 200 mg/dl up to the sacrifice day of the animals (p<0,0001, Figure 2B).

To evaluate islet functionality, a series of intraperitoneal glucose tolerance tests (IPGTT) were performed at different time points from transplantation and compared with healthy (n = 4) and diabetic (n = 4) reference curves (Figure 2C). The glucose clearance 15 days after transplantation showed that the decrease of glucose concentration in animals receiving 700 syn-IE plus 500,000 EPCs was observed 30 minutes after glucose administration unlike healthy animals (15 minutes); 120 minutes later there were no significant differences between the values. The curve relative to the co-transplanted group showed a very significant difference when compared with diabetic values (n = 4, p<0.001 120 min). Neither group of animals receiving 700 syn-IE alone (n = 4) nor EPCs alone (n = 3) showed a statistically significant difference from the diabetic group. Glucose-clearance of co-transplanted animals, at different time points, was compared to values of healthy animals (15, 30, 90 days after the transplant, Figure 2D). The trend in glucose clearance was very similar among the studied groups and it correlated well with the long-term control of glycaemic levels (Figure 2D).

### Neoangiogenesis triggered by EPCs in engrafted islets

**Figure 3:**
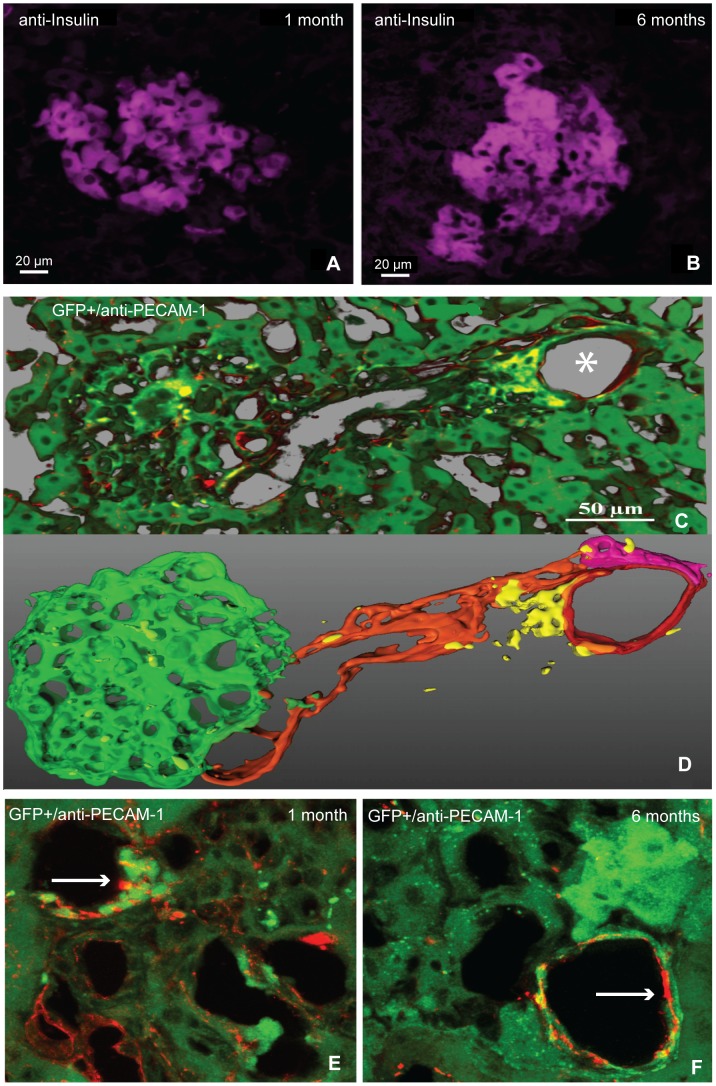
Histological assessment. Islet stained by anti-insulin antibody (magenta) respectively one month (A) and six months (B) after transplantation. (C–D) Islet, in the centre of a liver lobule, in contact with a network of new capillaries, as suggested by a strong presence of GFP+ EPCs (yellow) and PECAM-1 (red), from a perilobular vein (*) for animals at 30 days from transplant. The 3D reconstruction (D), better explain the previous picture (C). The islet was shown in green, while the perilobular vein was red. Orange represented the capillary network, the magenta an arteriole and the EPCs were yellow. New formed vessel in the islets one month (E) and six months (F) after transplantation, white arrows indicated endothelial (red) GFP+ EPCs. The endothelial cells in E exhibit a higher shape of those in F.

Livers and pancreata were excised for histological analysis to detect viable islets and the corresponding EPC neo-vascularization through insulin and PECAM-1 immunocytochemistry respectively (Figure 3). Transplanted islets were easily identified in liver samples by H&E staining and by anti-insulin immunofluorescence from one to six months after transplant (Figures 3A and B). PECAM-1 immunostaining, commonly used to demonstrate the presence of endothelial cells in histological tissue sections, when associated with the simultaneous presence of GFP+ EPCs demonstrates the presence of newly formed vessels in the grafts to derive blood flow from the host vessel system. In particular, in animals at 30 days from transplant, the new vessel appeared to emerge directly from the perilobular ones and EPC co-localization with PECAM-1 within the islets suggested newly formed blood vessels (Figures 3C and D). Furthermore, an average endothelial thickness of 4.77±1.1 microns one month after transplantation (Figure 3E) reinforced the notion of a juvenile cell as wall thickness retreats to 1.8±0.7 microns six months after transplant (Figure 3F), well within typical values of mature cells.

**Figure 4:**
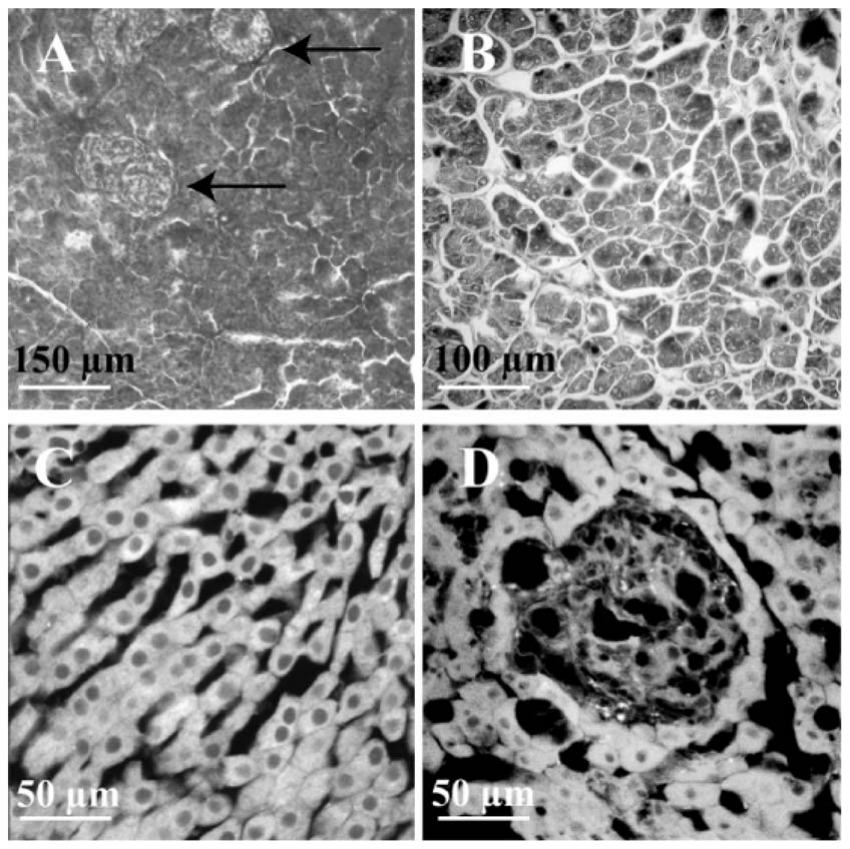
Evaluation of pancreas and liver tissues. (A) Viable islets in the pancreas of wild type animals as indicated by black arrows; (B) exocrine tissue without residual islets in a pancreas section of animals co-transplanted with GFP+ EPCs and wild type islets at 30 days after transplant. (C) Intact hepatic parenchyma of wild type animals; (D) liver tissue section of animals receiving GFP+ EPCs and wild type islets: image of a pancreatic islet at 30 days after transplant. No lymphocitic infiltration is evident.

Morphological assessment by H&E of pancreatic sections showed that no residual islets after STZ treatment were detectable. Comparison of the pancreatic tissue sections of wild type (Figure 4A) and co-transplanted EPC animals (Figure 4B) showed that islets were present only in wild type animals (black arrows), while the absence of islets was evident in the the exocrine pancreatic tissue of co-transplanted animals. Moreover by comparing the images of liver parenchyma of wild type animals (Figure 4 C) and animals transplanted with GFP+ EPCs and wild type islets (Figure 4D), we did not detect lymphocytic infiltration in the islet aggregates.

**Figure 5:**
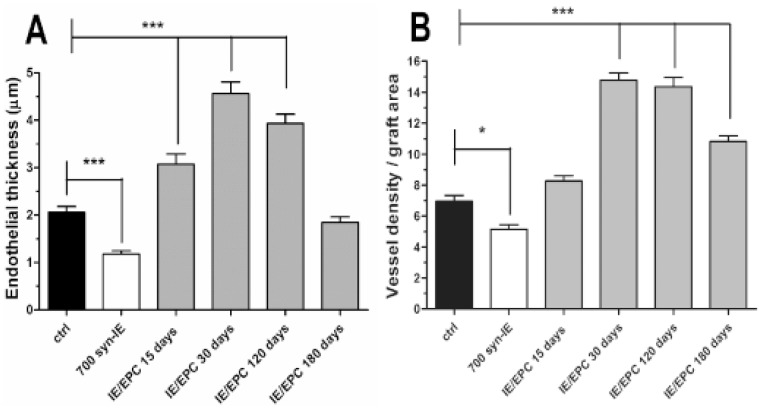
Vessel morphologic parameters assessment. (A) Endothelial thickness: average height(µm ± SEM) of the endothelial cells within the islets of wild type animals (black column), animals receiving islets alone (700 IE) (white column) and animals receiving 700 syn-IE plus 500,000 EPCs (IE/EPC) sacrificed at different time points (gray column)(***p<0.0001 at days 15, 30 and 120 versus control for IE/EPC group; ***p<0.0001 for 700 IE versus control). (B) Vessel density: average number (± SEM) of endothelial cells detected along a line in histological sections for wild-type animals, animals receiving islets alone (700 IE) (white column) and animals receiving 700 syn-IE plus 500,000 EPCs (IE/EPC) sacrificed at different time points (gray column) (***p<0.0001 at days 15, 30 and 120 versus control for IE/EPC group; ***p<0.0001 for 700 IE versus control).

We also evaluated whether an increase in the height of the endothelium and in the vessel density could result from EPC infusion in our model of islet transplantation (Figure 5). Both parameters were estimated by using stereological methods. Animals receiving 700 syn-IE plus 500,000 EPCs were sacrificed at different time points and were compared to both wild type control and animals receiving 700 syn-IE alone sacrificed at 30 days post-transplant.

Statistical differences in the height of the endothelium and vessel density were found among different groups. In particular, in co-transplanted animals, an increase of both parameters was observed in height (p<0.001) and density already at 15 days after transplantation and this trend increased up to 120 days (p<0.0001). At 180 days, the height of the epithelium was well within control values (Figure 5A), confirming typical thickness of a mature endothelium; on the contrary, the vessel density remained higher than control thus strengthening the already begun improved vascularization in accordance to a new vessel network formation (p<0.001) (Figure 5B).

Since the vascularization of the animals receiving islets alone (700 syn-IE) is highly compromised, the vascular epithelium appeared disrupted and consequently the endothelial thickness and vascular density were very low, under the control values (endothelial thickness p<0.001, Figure 5A; vessel density p<0.05, Figure 5B).

### *In vivo* EPC-induced regulation of angiogeneic genes

**Figure 6:**
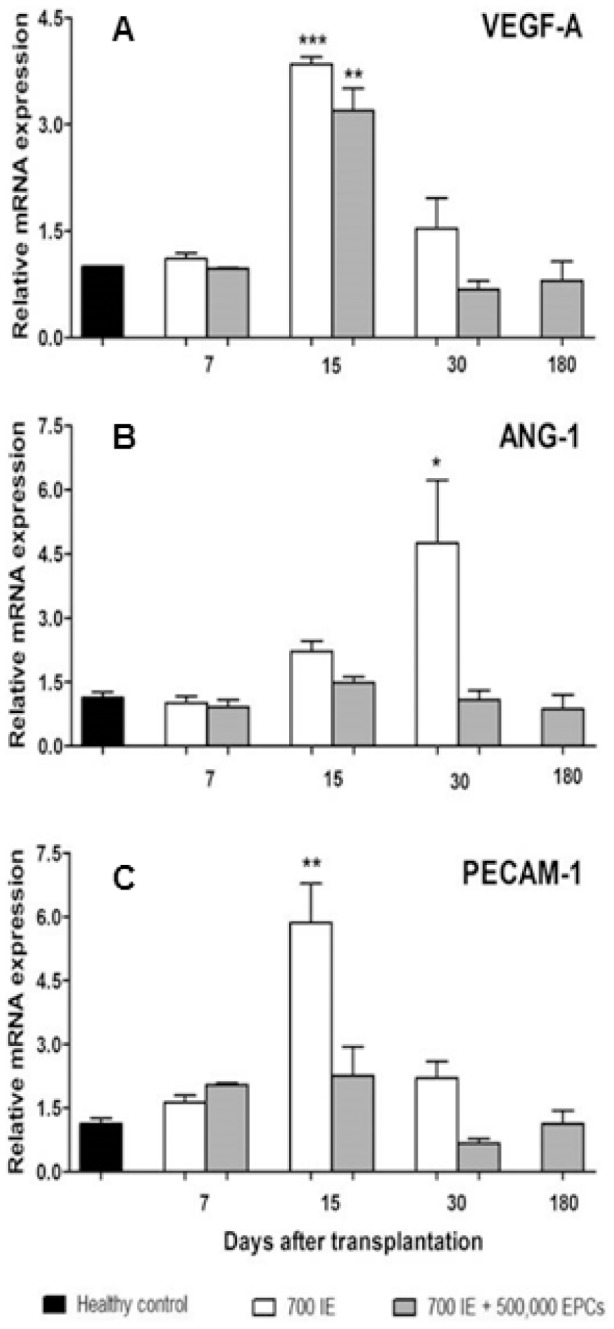
Modulation of angiogenic genes. mRNA levels of angiogenic factors in the livers of healthy animals (black bars), animals transplanted with 700 syn-IE (white bars) or 700 syn-IE+500,000 EPCs (grey bars) at different time points. (A) VEGF-A, ***p<0.0001 versus healthy. Compared to the control group the 700 syn-IE alone showed an higher increase (***p<0.0001) of VEGF-A expression than 700 syn-IE plus 500,000 EPCs(**p<0.001). Moreover statistical significant differences were found at different time point after transplantation inside the groups (700 syn-IE alone ***p<0.0001 day 7 versus day 15 and day 30 and ***p<0.0001 day 15 versus day 30; 700 syn-IE plus 500,000 EPCs **p<0.001 day 15 versus day 7 and day 30 and ***p<0.0001 day 15 versus day 180. (B) In ANG-1 expression no significant differences were found between the 700 syn-IE plus 500,000 EPCs group and healthy while at day 30 from transplant a significant increase was found in the 700 syn-IE alone group versus healthy (*p<0.01). Furthermore at day 30 a significant difference was found between 700 syn-IE alone and 700 syn-IE plus 500,000 EPCs one (**p = 0.0014). (C) PECAM-1, **p = 0.003 versus healthy. The column of gene expression relative to the animals treated with 700 syn-IE alone was not reported at 180 days, because the animals died within 45 days after transplantation due to lack of glycemic control. Error bars are +/− SEM.

To evaluate whether EPCs were able to modify angiogenic response through gene expression modulation, mRNA was extracted from the liver of transplanted animals and tested for VEGF-A, ANG-1and PECAM-1, specific genes involved in angiogenic response. Gene expression levels of animals transplanted with either 700 syn-IE alone or 700 syn-IE plus 500,000 EPCs were analyzed at different time points after transplantation (7, 15, 30 and 180 days) and compared to the expression in healthy controls. VEGF-A expression was maximal at day 15 from transplantation, followed by a significantly decrease at day 30 for both groups (Figure 6A). In relative ANG-1 mRNA expressions an increase was observed in animals receiving 700 syn-IE compared to the healthy group up to 30 post-transplant, (p<0.01), (Figure 6B). In contrast, the co-transplanted group did not show significant differences when compared to healthy controls. PECAM-1expression showed a markedly increase between 7 and 15 post-transplantation days in the 700 syn-IE group (p = 0.003), followed by a decrease at day 30 (Figure 6C). Conversely, the animals receiving co-transplantation showed significant differences compared to healthy control for the whole observation time.

## Discussion

Despite intense research carried out on EPC biology in the last 10 years a consensus on the definitive appearance and function of EPCs have not been yet reached [37]. Very little data are available on rat EPC characterization and at present a clear definition of EPC surface markers still remains elusive. Therefore there are controversial results obtained in EPC therapy derived from different EPC populations [39], [40]. I*n vitro*, two different populations of EPCs can be derived: early and late EPCs. Even if these cells are characterized by different morphology, proliferation potential and phenotype, *in vivo* these seem to improve vascularization [23]. In this work we studied the effect of bone marrow-derived rat late-EPCs in a marginal mass model of pancreatic islet transplantation in chemically-induced diabetic rats (STZ-treated), in an attempt to re-establish islet microvasculature destroyed during the islet isolation procedure. In a previous work we showed that transplantation of 700 IE in diabetic rat induced a significant decrease in blood glucose but failed to attain normoglycemia therefore this is considered a marginal mass model of islet transplantation [30]. We decided to transplant only a marginal mass of islets to overcome one of the main problems in islet transplantation: the limited donor-islet availability [41], [42]. On the basis of our results, we suppose that in the same model of full grafts (i.e. a transplantation of 1400 IE, [30]) the EPCs would be able to support islet revascularization better than in the marginal mass model. It is well known in fact that long-lasting hyperglicemia impairs the vascular network [14] preventing the revascularization process. In the full graft model normoglycemia is reached faster and for a long period than in the marginal mass, thus the EPC efficacy in the islet revascularization could be increased. Our results highlight that neo-vascularization is a crucial process in promoting a viable and enduring islet pancreatic transplant in experimental diabetes. GFP+ EPCs that lasted for 180 days after co-transplant promoted a newly formed vascular network. Accordingly, histological analysis revealed insulin-positive clusters of islets surrounded by GFP-expressing newly formed endothelial cells only in livers of recipients that received a co-transplant. This was combined with a long-lasting normoglycemia and a modulation of the expression of angiogenesis-related genes, which accompanied a new blood vessel formation. Since we did not detect any residual beta cell mass in pancreatic tissues of STZ-treated animals by H&E analysis, we speculated that the role of EPCs was to sustain the function of donor grafted islets more than residual islets of the recipients. Our data is in accordance with recently published results by Oh *et al*. They showed that not only donor EPC co-transplanted with islets are able to improve intra-islet microvasculature but also contribute to maintain islet organization and morphology. Unlike us they used early EPCs co-transplanted with a marginal mass of syngenic islets under kidney capsule of diabetic-induced mice [43]. Impaired revascularization is one of the main issues of graft failure [44] and attempts have been made to overcome this obstacle through administration of angiogenic factors such as VEGF [45] and/or mesenchymal stem cells [46], or stimulation with GM-CSF to mobilize bone marrow-derived EPCs [47]. Nevertheless angiogeneic factors had a short half-life as consequence their use is not free of safety concerns [48]. On the other side the migration of mesenchymal stem cells towards the site of inflammation and their dispersion in several organs of the recipient [49]–[53] reduced their efficacy on the grafted islets and could increase the risk of tumour development [54], even though recently a pooled analysis showed no correlation between MSCs and malignancies [55]. GM-CSF treatment could be appealing for its immediate clinical translational potential, on the other hand this approach might have significant effects on the immune system of the recipient [47]. In this context the interest towards the effect of EPCs in islet transplantation arised; in 2004, Brissova *et al*. reported that *in vitro* EPCs co-cultured with pancreatic islets were able to improve beta cell survival and insulin secretion [56] and recently many works on EPC and islet co-transplantation have been published [27], [43], [57]. The EPC-cotransplant method described here could be a more physiological way of inducing neovascularization in islet transplant. EPCs in fact did not disperse into the recipient but were confined in the implantation site around the transplanted islets thus making them safe for use in clinical settings. Overall EPCs were found to support beta cell proliferation [56], [58], cause a threefold improvement of beta cell volume and double functional blood vessels [57]. In addition, it has to be considered that patients with diabetes are characterized by low levels of circulating EPCs correlated to the impaired endothelium, so that the revascularization process is delayed upon an ischaemic insult (one week later compared to the healthy patient) [16], [59]. A re-establishment of a complete vascular network was successfully observed in the present study. Most importantly, the overlapping of blood glucose levels, blood glucose control and revascularization lasting 180 days suggests that donor EPCs may play a key role in enhancing and maintaining revascularization over long periods of time. Many works have also investigated the structure and function of intra-islet endothelial cells to clarify their role in blood vessel regeneration and in revascularization of islet graft [56], [60], [61]. Nyqvist *et al.* observed that the transplantation of freshly isolated islets with a relevant number of endothelial cells, in contrast to cultured islets, markedly improved their vascularization, thus a preservation of intra-islet endothelial cell mass was able to improve the long-term graft function [62]. Later the same authors further observed that donor islet endothelial cells contributed to the revascularization of freshly isolated islets by participating in early processes of vessel formation; nevertheless, these cells did not increase the vascular density or improve the endocrine function of the grafts [63]. In our work the absence of residual islets of the recipient in co-transplanted animals after STZ treatment supports the hypothesis that normoglycemia is due to donor islets and EPCs. Donor EPCs could effectively contribute to the intra-islet EPCs to support islet function and maintain morphological organization. Furthermore, the induced neovascularization in the co-transplanted group by EPCs is explained by the modulation of specific gene expression involved in the angiogenic process, whereas this is not observed in the control group. VEGF is the most important gene involved in the regulation of blood vessel sprouting during development, growth and disease; in particular, VEGF-A member is positively regulated by hypoxia [64]. Our data show a marked increase in the VEGF-A level in liver tissues of animals in the first 15 days after transplantation for groups receiving both 700 syn-IE and 700 syn-IE plus EPCs. It has to be considered that islets secrete VEGF-A after isolation, as the result of the ischemic insult derived from it and from the culture condition and also that in the first days after transplantation there is an incomplete vascular network formation and only a partial recovery in functionality. Nevertheless in the co-transplanted group the increase in VEGF-A expression was lower than in the group of animals receiving islet alone, due to an exogenous administration of EPCs. Our findings are in line with data demonstrating that the vascularization of transplanted islets is delayed by the presence of hyperglycemia, derived from an increase in local oxygen consumption [65]. ANG-1 is responsible for vessel stabilization and promotion of pericytes adhesion by tightening endothelial junctions [66]. Jeansson *et al.* demonstrated that ANG-1 is not only necessary in the quiescent mature vasculature, but it also exerts a role in the regulation of the response to tissue injury and microvascular disease in diabetes [67]. High levels of ANG-1 gene expression, observed in the 700 syn-IE group, suggest that islets were unable to tighten endothelial junctions and maintain blood vessels in host diabetic environment. On the contrary, the down-regulation of ANG-1 observed in the co-transplanted group, is probably related to the EPC-supported vascularization. At variance with the other genes, PECAM-1 is involved in transendothelial migration of neutrophils, monocytes and natural killer cells both *in vitro* and *in vivo*. Indeed transmigration and inflammation can be significantly reduced when antibodies directed against PECAM-1 are used [68]. The strong down-regulation of PECAM-1 in co-transplanted group indicates that administration of exogenous EPCs is effective also in reducing the recruitment of immune system cells thus hampering an inflammatory response as shown by the absence of infiltrating mononuclear cells into the liver parenchyma. This result was supported also by Cantaluppi *et al.* who demonstrated that microvesicles, released by EPCs on human pancreatic islets, significantly inhibited spontaneous and cytokine-induced peripheral blood mononuclear cell adhesion to islet endothelial cells [69]. Microvescicles expressed CD154 marker as able to bind CD40 expressed by islet endothelial cells, thus interfering with leukocyte adhesion to endothelium. Overall our obtained results by gene expression suggest that day 15 is the crucial time point in graft revascularization. Recently Kang *et al.* reported that human cord blood-derived EPCs co-transplanted with porcine islets into renal capsules of diabetic nude mice were able to induce a rapid revascularization, a better graft perfusion and a recovery from hypoxia [27]. Although this experimental design was similar in some respects to our transplantation model, there were some differences in the implantation site, the origin of both islets and EPCs, timing of analysis and the islet dose used. To investigate the effect of EPCs on islet vascularization either early or a long time after transplantation, we chose a syngenic transplantation model to avoid any other interferences due to allo- or xenograft rejection. Moreover, the kidney subcapsular site is most commonly used in rodent models and has good results in that diabetes is reversed within a few days. Nevertheless, though the development of an instant blood-mediated inflammatory reaction (IBMIR) upon intraportal islet infusion, the progressive loss of islet function even in recipients of autologous grafts (in humans, but also in the canine model [70]), the portal vein implantation site still remains the gold standard for islet transplantation in the clinical setting. Until now the intraportal site was considered to have a reduced islet survival, not suitable for long-term function [70], while the long-term normoglycemia that we observed highlights a fully revascularization and a complete functionality of engrafted islets even when a marginal mass was used. A limit of our study is the assessment of pancreatic islet transplant only in a syngenic rat model to avoid an immunologic response due to donor-recipient immunologic systems. This needs certainly to be further investigated in the next future in an allogeneic co-transplant model, also by exploiting other cell lines like Sertoli cells which appeared to maintain the immunosuppressive effect during vascularization process [71] or either protocols of immunosuppression [72]. This could overcome obstacles deriving from the host's immune rejection and impairment of vascularization network in the long term. An interesting issue on the mechanism of islet revascularization involves the emerging measurements of vessel parameters within the endothelial lumen. As reported by Alberts *et al*., the endothelial cells not only repair and renew the lining of established blood vessels, but also create new blood vessels and have a remarkable capacity to adjust their number and arrangement to suit local requirements [73]. By signaling to the surrounding cells, endothelial cells enable the blood vessel to adapt its diameter and wall thickness to suit the blood flow needed. They can be roused to proliferate with a doubling time of just a few days [73], including variations of thickness as it occurs in the allogeneic portal vein transplants, that developed a significant increase in wall thickness [74]. Recently several studies of co-transplant of pancreatic islets and EPCs reported conflicting results about the effect of EPCs on vascular density and their role in the mechanism of improvement in vascularization process [27], [57]. Our results demonstrated that EPCs were able to increase the vascular density and the endothelial thickness in co-transplant model during the first 30 days after transplantation. Subsequently a decrease of both parameters was observed up to levels comparable with healthy control values. This effect can be attributed to the endothelium transition from a juvenile to a mature status. It is worthy of note that few data is present in the literature about endothelial thickness which for the first time was analyzed in a study about the revascularization process. In conclusion, we provide evidence that co-transplantation of EPCs and a marginal mass of pancreatic islets in portal vein induce a stable rescue of glycemic control lasting for a significant fraction of the animal life span. We show that the glycemic control recovery is associated with EPC-induced neovascularization, which is followed by a stabilization of islet vascular network within a few weeks after transplantation. This is paralleled by a down-regulation of specific genes, such as ANG-1, involved in the vascularization process, and PECAM-1 related to the inflammation process. This provides the experimental evidence for the previously hypothesized revascularization process as a key factor for successful islet transplantation. The present results pave the way to translational experimental testing in humans as a new therapeutic approach to overcome some problems encountered in the search for a successful and long-lasting surgical approach for the cure of IDDM.

## Supporting Information

Figure S1 **Time lapse of EPCs/GFP+ EPCs onto BD Matrigel Basement.** (A, B, C) EPCs shown an excellent ability to form a capillary-like structure (respectively, 8 hours, 12 hours, 20 hours). (D, E, F) Bright field images of GFP+ EPCs and (G, H, I) fluorescence images show that GFP+ EPCs grow gradually from a sparsely scattered capillary structure (2 hours after seeding, D–G) to a more organized (4 hours after seeding, E–H) and finally to a complete capillary network (6 hours after seeding, F–I).
(TIF)
