# Supplementary figures and images for: Co-Transplantation of Endothelial Progenitor Cells and Pancreatic Islets to Induce Long-Lasting Normoglycemia in Streptozotocin-Treated Diabetic Rats
Source: PLoS One. 2014 Apr 14;9(4):e94783. doi: 10.1371/journal.pone.0094783 (PMC3986409; doi:10.1371/journal.pone.0094783)

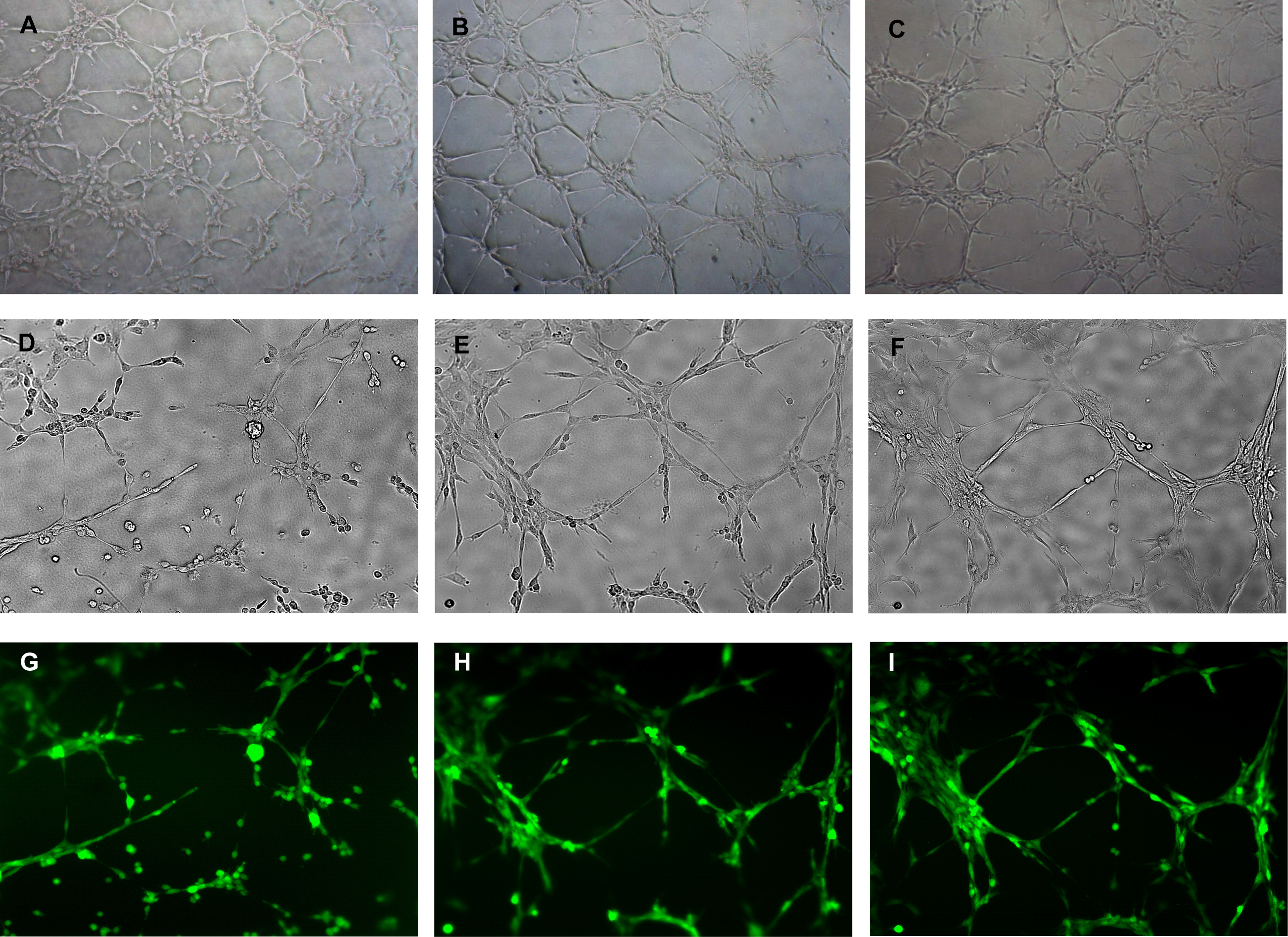

Supplement: Figure S1 — Time lapse of EPCs/GFP+ EPCs onto BD Matrigel Basement. (A, B, C) EPCs shown an excellent ability to form a capillary-like structure (respectively, 8 hours, 12 hours, 20 hours). (D, E, F) Bright field images of GFP+ EPCs and (G, H, I) fluorescence images show that GFP+ EPCs grow gradually from a sparsely scattered capillary structure (2 hours after seeding, D–G) to a more organized (4 hours after seeding, E–H) and finally to a complete capillary network (6 hours after seeding, F–I). (TIF) [file pone.0094783.s001.tif]
